# Health Information Systems: how much progress are we making?

**DOI:** 10.1590/S2237-96222023000200001

**Published:** 2023-08-21

**Authors:** Barbara Reis-Santos

**Affiliations:** 1 Rede Brasileira de Pesquisas em Tuberculose, Rio de Janeiro, RJ, Brasil

The development and consolidation of the Brazilian National Health System (*Sistema Único de Saúde* - SUS) are at the heart of the establishment of Health Information Systems (HIS) nationwide. Although some records predate the SUS, it was based on its structuring that the legal and operational framework for the systematic collection of data evolved, both for administrative purposes and for monitoring diseases and health conditons.^1^ In programmatic terms, HIS data can be the basis for structuring public health actions and planning and evaluating programs; in the academic context, they contribute to the formulation and implementation of research hypotheses.^2^ ^),(^ ^3^ HIS in Brazil are an important source of structured data for researchers, as discussed in a recent editorial^4^ of the Epidemiology and Health Services journal (revista *Epidemiologia e Serviços de Saúde* - RESS) on population data science, which can also be noted by their constant presence in articles published in the journal - there are sixteen such articles in this issue.

The heterogeneous nature of the way these databases are constituted is a challenge for the professionals responsible for their maintenance and management and, moreover, raises research questions for researchers who work with the subject. Evaluation of the quality and sensitivity of Brazilian HIS data is a recurrent object of research published in the RESS and is present in this issue of the journal.^5^ ^)-(^ ^9^ The importance of producing solid evidence on HIS performance is undeniable; it is however reasonable to ask how much progress has been made in relation to the development of other aspects of these systems.

Before doing so, when attempting to answer an earlier question about the number of nationally based HIS existing in Brazil, Coelho Neto et al. identified fifty-four in operation between 2010 and 2018.^2^ Even with this extensive list, it is possible that professionals and researchers still feel the lack of one or more databases with which they work did, which reinforces the authors’ conclusion about the need to promote transparency in the management of information technology policy within the scope of the Ministry of Health in order to achieve effective accountability.^2^

Today, the website of the Health Ministry’s Health and Environmental Surveillance Secretariat has an HIS tab which lists the Live Birth Information System (*Sistema de Informações sobre Nascidos Vivos* - SINASC), the Notifiable Health Conditions Information System (*Sistema de Informação de Agravos de Notificação* - SINAN), the e-SUS Notifica system, the Public Health Event Registry - Microcephaly (*Registro de Eventos em Saúde Pública - Microcefalia: RESP-Microcefalia*) and the Mortality Information System (*Sistema de Informações sobre Mortalidade* - SIM), which are related to the surveillance of chronic non-communicable diseases.^10^ In turn, thirty datasets can be found on the *openDATASUS* webpage, derived from the following systems: e-SUS Notifica, the National Immunization Program Information System (*Sistema de Informação do Programa Nacional de Imunizações* - SI-PNI), the National Health Establishment Registry (*Cadastro Nacional de Estabelecimentos de Saúde* - CNES), the Influenza Epidemiological Surveillance Information System (*Sistema de Informação da Vigilância Epidemiológica da Gripe* - SIVEP-Gripe), the Quality of Water for Human Consumption Surveillance Information System (*Sistema de Informação de Vigilância da Qualidade da Água para Consumo Humano* - SISAGUA), the Food and Nutrition Surveillance System (*Sistema de Vigilância Alimentar e Nutricional* - SISVAN), the SIM system, the SINASC system, the COVID-19 Platform, Indigenous Health Care Special Secretariat (*Secretaria Especial de Saúde Indígena* - SESAI), the Indigenous Health Care Information System (*Sistema de Informações da Atenção à Saúde Indígena* - SIASI), the SINAN system and the Sylvan Health Information System (*Sistema de Informação em Saúde Silvestre* - SISS-Geo).^11^ With the aim of presenting the Brazilian databases of interest for epidemiology, disease surveillance, prevention and control, with effect from 2017 the RESS has published manuscripts in the National Health Database Profile category.^12^ ^)-(^ ^17^ Additionally, experiences of Brazilian population data centers, in Belo Horizonte, Salvador and Rio de Janeiro, have been recognized for their work with Brazilian HIS, especially for their contribution to linkage between the different databases.^18^

The initiatives listed above are important, but are insufficient to maintain progress in the area. Making public, and on an ongoing basis, the complete list of available HIS, the associated metadata and access to their anonymized microdata is fundamental for effective accountability.^2^ ^),(^ ^4^ ^),(^ ^19^ Development efforts for updating and integrating the different systems, so that they take into account the speed of health responses, as well as the establishment of rules that minimize inconsistencies in the databases, are also necessary forms of progress. More in-depth analyses of the systems’ various attributes, such as usefulness, simplicity, flexibility, data quality, acceptability, sensitivity, representativeness, timeliness, and stability, are equally important.^3^

Over the years the HIS managed by the Brazilian Ministry of Health have become internationally recognized for their robustness and reliability. However, during the COVID-19 pandemic, lack of timeliness in accessing information contributed to these systems taking a secondary role, so that media vehicles and researchers began to seek other sources to achieve timely disclosure of cases and deaths, with the latter even being accessed by civil registries. In the same period, the Ministry of Health launched an information system to meet the response needs of the pandemic. In this context, the establishment of a coordinated and continuous set of actions that promote HIS transparency and accountability can collaborate with the continuing evolution of these systems, so that they are more sensitive for detecting changes in the behavior of diseases and health conditions, in addition to contributing to addressing new pandemics.
